# Heavy burden of non-communicable diseases at early age and gender disparities in an adult population of Burkina Faso: world health survey

**DOI:** 10.1186/1471-2458-12-24

**Published:** 2012-01-10

**Authors:** Malgorzata Miszkurka, Slim Haddad, Étienne V Langlois, Ellen E Freeman, Seni Kouanda, Maria Victoria Zunzunegui

**Affiliations:** 1 Department of Social and Preventive Medicine, University of Montreal, Montreal, Canada; 2 Research Centre of the University of Montreal Hospital Centre, Montreal, Canada; 3 Research Centre, Maisonneuve-Rosemont Hospital, Montreal, Canada; 4 Department of Ophthalmology, University of Montreal, Montreal, Canada; 5 Institut de Recherche en Sciences de la Santé (IRSS), Ouagadougou, Burkina Faso

## Abstract

**Background:**

WHO estimates suggest that age-specific death rates from non-communicable diseases are higher in sub-Saharan Africa than in high-income countries. The objectives of this study were to examine, in Burkina Faso, the prevalence of non-communicable disease symptoms by age, gender, socioeconomic group and setting (rural/urban), and to assess gender and socioeconomic inequalities in the prevalence of these symptoms.

**Methods:**

We obtained data from the Burkina Faso World Health Survey, which was conducted in an adult population (18 years and over) with a high response rate (4822/4880 selected individuals). The survey used a multi-stage stratified random cluster sampling strategy to identify participants. The survey collected information on socio-demographic and economic characteristics, as well as data on symptoms of a variety of health conditions. Our study focused on joint disease, back pain, angina pectoris, and asthma. We estimated prevalence correcting for the sampling design. We used multiple Poisson regression to estimate associations between non-communicable disease symptoms, gender, socioeconomic status and setting.

**Results:**

The overall crude prevalence and 95% confidence intervals (CI) were: 16.2% [13.5; 19.2] for joint disease, 24% [21.5; 26.6] for back pain, 17.9% [15.8; 20.2] for angina pectoris, and 11.6% [9.5; 14.2] for asthma. Consistent relationships between age and the prevalence of non-communicable disease symptoms were observed in both men and women from rural and urban settings. There was markedly high prevalence in all conditions studied, starting with young adults. Women presented higher prevalence rates of symptoms than men for all conditions: prevalence ratios and 95% CIs were 1.20 [1.01; 1.43] for joint disease, 1.42 [1.21; 1.66] for back pain, 1.68 [1.39; 2.04] for angina pectoris, and 1.28 [0.99; 1.65] for asthma. Housewives and unemployed women had the highest prevalence rates of non-communicable disease symptoms.

**Conclusions:**

Our work suggests that social inequality extends into the distribution of non-communicable diseases among social groups and supports the thesis of a differential vulnerability in Burkinabè women. It raises the possibility of an abnormally high rate of premature morbidity that could manifest as a form of premature aging in the adult population. Increased prevention, screening and treatment are needed in Burkina Faso to address high prevalence and gender inequalities in non-communicable diseases.

## Background

The steady rise in non-communicable diseases (NCDs) worldwide is a key challenge on the global health agenda. Not only are chronic pathologies the leading cause of mortality globally, but they represent an increasing burden of morbidity and mortality in the developing world [1]. World Health Organization (WHO) estimates show that age-specific death rates from non-communicable diseases are already higher in sub-Saharan Africa (SSA) than in established market economies [2]. Moreover, overall mortality rates are higher in low- and middle-income countries (LMIC) than in high-income countries (HIC) [3].

NCDs increasingly take their toll in Western Africa in the form of cardiovascular diseases and pulmonary pathologies. The prevalence of asthma on the African continent has been estimated at 10.4% [3,4], and a similar rate of 9.6% was observed in a population aged 15 to 64 years in the urban setting of Bobo-Dioulasso, Burkina Faso [5]. The prevalence of hypertension in an urban setting of Burkina Faso was estimated at 23% among adults aged 18 years or older [6]. The rise in NCDs has been attributed to rapid urbanization, sedentary lifestyles, global marketing of tobacco and food, and population aging [7]. In fact, economic growth, market integration, foreign direct investments and urbanization are driving long-term changes in mortality due to cardiovascular diseases and other NCDs, and their influence in LMICs is roughly three times stronger than that of population aging [8].

In spite of worldwide recognition that NCDs are a growing health problem that will hinder LMICs' social development and economic growth, few population-based studies are available to estimate their prevalence and distribution by age, gender, social group or urban/rural setting. In addition, while the literature clearly describes social differentials in health and presents conclusive and repeated evidence that the burden of illness falls disproportionately on women and the poor [9-12], little is known about the scope of these inequalities with respect to NCDs, particularly in sub-Saharan Africa. Because women have less wealth, are more likely to be uneducated and have higher burdens of work [13,14], all deriving from structures that shape gender and economic inequality, we can also expect to observe gender-related disparities, with higher prevalence of NCDs in women than in men. In fact, in 2004, the estimated proportional mortality from all NCDs in Burkina Faso was 24.7% for men and 27.6% for women [15]. Drawing upon data on Burkina Faso in the World Health Survey (WHS), a population-based household survey, our aim in this study was to describe the prevalence of NCD symptoms by age, gender, socioeconomic group and rural/urban setting, and to assess gender and socioeconomic differences in the prevalence of these symptoms.

## Methods

### Sample

We obtained data from the WHS conducted by the WHO and local collaborative partners in all world regions starting in 2001 (available from WHO on request). Of the 70 countries surveyed, 18 were in the African region. Our study focused on the eligible adult population (18 years and over) of Burkina Faso. The WHS used multi-stage stratified random cluster sampling strategy to identify participants. Their sampling strategy consisted of stratifying the population by gender, age and setting (rural/urban). Individuals were sampled from these strata, and each respondent was given a sampling probability value. Using a Kish table, one adult 18 years or older was selected from each randomly selected household to undergo an individual, face-to-face interview using a standardized questionnaire. Questions were aimed at identifying socio-demographic and economic characteristics as well as symptoms of chronic diseases, including joint disease, back pain, angina pectoris and asthma. Informed consent was obtained from all participants. The individual response rate was 98%, and the highest percentage of missing data for an individual variable was 0.7%, leaving us with a sample of 4822 participants for the final analysis.

Four outcome variables were identified based on chronic disease items from the WHS. The questionnaire included 46 NCD-related questions investigating symptoms and known diagnoses of NCD. The use of symptom questions for diagnosing chronic diseases was previously validated in a diagnostic item probability study implemented in 2003 by WHO in seven countries [16].

### Outcomes

We selected the reported symptoms corresponding to four NCD conditions: joint disease, back pain, angina pectoris and asthma. These conditions are all associated with lower quality of life, high medical and non-medical care costs, and loss of productivity, and together they account for a large proportion of mortality [1-3,17,18]. We chose these diseases because, unlike diabetes, for instance, they have specific and more easily recognizable symptoms that are useful to estimate the burden of disease in the absence of medical diagnoses. We excluded important and frequent non-communicable disease symptomatologies such as hypertension and diabetes from the WHS because of the lack of specific symptoms in the earlier stages of disease. For the four conditions studied, participants were asked if they had experienced symptoms during the past 12 months, such as, in the case of joint disease: pain, aching, stiffness or swelling in or around a joint not related to injury and lasting for more than a month (yes/no). Answers for all four NCD symptoms (joint disease, back pain, angina pectoris and asthma) were dichotomized as the presence or absence of self-reported symptoms.

### Variables

Gender, age (18-24, 25-34, 35-44, 45-54, 55-64, 65 years and older) and rural or urban setting were the main socio-demographic variables of interest. Three socioeconomic characteristics were determined by the respondents: 1) education (lack of schooling; some formal schooling); 2) occupation, defined as unemployed, homemaker/caring for family, agriculture/fishery worker, manual worker (machine operator, service or sale, craft or trades, street vendor), or non-manual worker; and 3) annual household income, based on self-reported expenditure on food, housing, health care costs and all other goods and services in the past four weeks. Income was approximated by household annual consumption per capita. We used the poverty line (PL) for Burkina Faso in 2003 [19] to create four income categories: 1) poorest, with annual income less than one-half of the poverty line (< PL/2); 2) below the poverty line but above the poorest (≥ PL/2, < PL); 3) above, but less than double, the poverty line (≥ PL, < 2 × PL); and 4) more than double the poverty line (≥ 2 × PL).

### Statistical analysis

First we examined the distribution of the socio-demographic and economic variables by gender and by urban and rural settings. We then estimated the crude prevalence and 95% confidence intervals (CI) for each of the NCDs and reported them by age, gender and setting. Crude prevalence ratios and chi-squared test results were also reported according to socioeconomic groups. Next, we constructed Poisson regression models [20] to examine the prevalence ratios of NCDs according to gender. We created a crude model and then successive full models. Socioeconomic variables were included in models by set, as explanatory variables for gender-health relationships. We tested all interactions of gender with socioeconomic variables to assess possible differential vulnerability to NCD symptoms by gender according to social conditions. The final stage of analysis consisted of four sets of Poisson regression models for each set of symptoms as the outcome.

Model building was preceded by including all independent variables that were univariate predictors of NCD symptomatology at p-value ≤ 0.05. In a subsequent step, variables that were not predictors were entered into the final model one at a time and retained as multivariate predictors if p ≤ 0.05. Reported p-values of ≤ 0.05 were considered statistically significant. Analyses were conducted with Stata statistical software (version 11) where individual data were corrected for the complex sampling design using the survey commands for weights.

## Results

### Sample characteristics and prevalence rates

**Figure 1:**
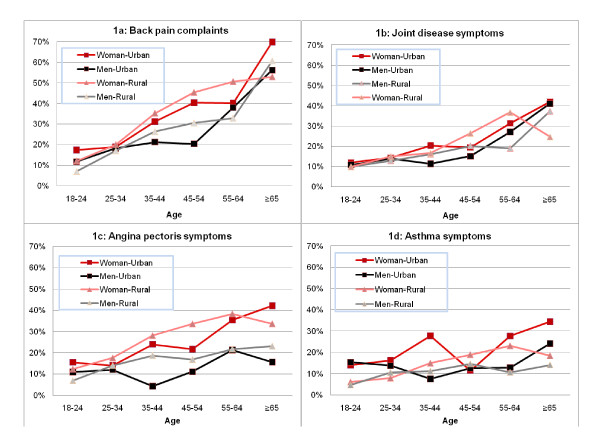
**Crude prevalence of self reported symptoms of chronic disease by age in men and women in urban and rural settings**.

The demographic and socioeconomic characteristics of the sample are presented in Table 1. The youngest group, 18-24 years old, was more concentrated in urban settings, with a higher concentration of women (30.6%) than men (23.4%). Homemaker was the main occupation for women, whereas manual worker or agriculture/fishery worker was most common for men. The overall crude prevalence and 95% CI for NCD symptoms were: 16.2% [13.5; 19.2] for joint disease, 24% [21.5; 26.6] for back pain, 17.9% [15.8; 20.2] for angina pectoris, and 11.6% [9.5; 14.2] for asthma. Higher prevalence of symptoms of joint disease, back pain, and angina pectoris were reported in the rural population, whereas asthma symptoms were more prevalent in the urban population (Figure 1). Table 2 presents the crude prevalence of NCD symptoms across social groups. Back pain symptoms were more often reported by educated respondents, whereas asthma symptoms were more prevalent among the non-educated. A convergent pattern was observed across occupational groups: a health gradient was noticed for all symptoms, with the highest prevalence rates being observed among the unemployed and homemakers. No clear pattern emerged from the study of the association between crude prevalence rates and household income.

**Table 1:** Demographic and socioeconomic characteristics by gender in urban and rural settings for an adult (≥ 18 years old) population of Burkina Faso (n = 4822) (WHS 2003).

Socioeconomic variables (%)	Urban		Rural	
	
	Women	Men	Women	Men
***Age***	*n = 1031*	*n = 898*	*n = 1519*	*n = 1374*
18-24	30.6	23.4	26.7	20.7
25-34	29.7	28.8	30.5	28.6
35-44	19.3	23.3	18.7	19.6
45-54	10.3	13.7	11.3	11.8
55-64	5.7	5.8	7.3	8.9
65 and older	4.4	5.0	5.5	10.5
***Marital status***	*n = 1031*	*n = 897*	*n = 1518*	*n = 1374*

Widowed	11.0	1.1	8.4	2.2
Separated/divorced	13.6	31.3	2.8	15.5
Single	2.6	1.9	1.4	1.8
Married	72.8	65.7	87.4	80.5
***Education***	*n = 1031*	*n = 898*	*n = 1519*	*n = 1374*

No formal education	59.3	46.0	95.3	87.8
Some formal education	40.7	54.0	4.7	12.2
***Occupation***	*n = 1031*	*n = 898*	*n = 1519*	*n = 1374*

Unemployed	11.7	11.9	6.8	11.0
Homemaker	41.7	0.3	60.4	1.2
Agriculture or Fishery	7.4	18.0	23.8	77.5
Manual worker	28.1	45.8	8.3	8.1
Non-manual worker	11.1	23.9	0.7	2.2
***Annual household income***	*n = 1021*	*n = 887*	*n = 1507*	*n = 1357*

< PL/2	11.2	11.3	38.0	33.9
≥ PL/2-< PL	26.9	25.1	36.1	33.2
≥ PL-< 2 × PL	35.3	33.8	20.4	23.1
≥ 2 × PL	26.6	29.8	5.4	9.9

PL corresponds to the poverty line for Burkina Faso in 2003, which was 82,672 Franc CFA/year [17] (< PL/2): poorest, with annual income less than one-half of the poverty line; (≥ PL/2, < PL): below the poverty line but above the poorest; (≥ PL < 2 × PL): above, but less than double, the poverty line; (≥ 2 × PL): more than double the poverty line.

**Table 2:** Crude prevalence rates for non-communicable disease symptoms by socioeconomic groups in an adult (≥ 18 years) population of Burkina Faso (WHS 2003).

	Joint Disease	Back Pain	Angina Pectoris	Asthma
**Education**				
No formal education	16.2 [13.3; 19.6]	25 [22.2; 28.1]	18.6 [16.3; 21.1]	11 [8.75; 13.6]
Some formal education	16.1 [12.7; 20.2]	18 [14.7; 21.7]	14.6 [10.8; 19.4]	15.4 [11.4; 20.4]
	*P = 0.9547*	*P = 0.0024*	*P = 0.1037*	*P = 0.0339*

**Occupation**				
Unemployed	21 [16.6; 26.1]	32.4 [26.9; 38.3]	25.1 [19.5; 31.6]	17.8 [13.7; 22.9]
Homemaker	19.8 [15.9; 24.4]	28.3 [24.0; 33.1]	20.3 [16.8; 24.3]	13.3 [10.0; 17.3]
Agriculture or Fishery	14.1 [10.9; 18.2]	20.4 [17.4; 23.7]	16.0 [14.0; 19.2]	9.4 [6.9; 12.5]
Manual worker	11.7 [8.7; 15.6]	20.8 [16.5; 26.0]	14.2 [10.8; 18.5]	9.51 [6.6; 13.4]
Non-manual worker	12.7 [8.4; 18.8]	14.7 [9.4; 22.2]	10.6 [6.6; 16.7]	14.4 [8.8; 22.7]
	*P = 0.0007*	*P = 0.0000*	*P = 0.0006*	*P = 0.0042*

**Annual household income**				
< PL/2	15.8 [12.7;19.3]	22.5 [19.0;26.4]	16.8 [14.1;19.9]	10.4 [7.9;13.6]
≥ PL/2-< PL	16.1 [13.1;19.5]	25.7 [22.1;29.7]	19.7 [16.3;23.5]	11.3 [8.2; 15.3]
≥ PL-< 2 × PL	16.8 [12.5;22.1]	23 [19.7; 26.5]	17.7 [14.5; 21.4]	12.7 [10.4; 15.4]
≥ 2 × PL	17 [12.6; 22.5]	24.6 [20.0; 29.9]	16.6 [13.2; 20.7]	15.3 [10.8; 21.2]
	*P = 0.9268*	*P = 0.3954*	*P = 0.3961*	*P = 0.1957*

Note: p-values correspond to design-based chi-squared test results.

### Gender gaps and early onset of NCD symptoms

Independent of setting, women reported more symptoms than men for all four NCDs. This difference was more pronounced in middle-aged groups, especially for back pain and angina pectoris symptoms. Prevalence rates of over 5% for all NCD symptoms were reported even in the youngest population of 18-24 years old (Figure 1) and increased steeply at middle age, affecting the most economically productive population.

Women were more apt than men to report symptoms for all four NCDs, with the greatest difference seen in symptoms of angina pectoris (prevalence ratio = 1.68; CI = 1.39-2.04). The prevalence ratio for angina pectoris remained almost unchanged after the inclusion of socioeconomic factors (OR = 1.64; CI = 1.39-1.94) (Table 3).

**Table 3:** Prevalence ratios (PR) and confidence intervals (95%) for non-communicable disease symptoms in an adult (≥ 18 years) population of Burkina Faso (WHS 2003).

	Joint Disease		Back Pain		Angina Pectoris		Asthma	
	
	Crude model	Full model	Crude model	Full model	Crude model	Full model	Crude model	Full model
**Gender (reference category = Men)**								
Women	1.20* [1.01;1.43]	1.18 [1.00;1.40]	1.42* [1.21;1.66]	1.27* [1.09;1.47]	1.68* [1.39;2.04]	1.64* [1.39;1.94]	1.28 [0.99;1.65]	1.34* [1.05;1.70]

**Age (reference category = 18-24 years old)**								
25-34		1.44* [1.11;1.87]		1.61* [1.27;2.04]		1.60* [1.23;2.07]		1.58 [1.14;2.20]
35-44		1.73* [1.29;2.33]		2.57* [2.00;3.30]		2.37* [1.75;3.22]		2.40* [1.58;3.64]
45-54		2.38* [1.80;3.15]		3.18* [2.35;4.30]		2.55* [1.81;3.60]		2.65* [1.67;4.22]
55-64		3.15* [2.04;4.86]		3.57* 2.61;4.88]		3.07* [2.14;4.39]		3.05* [1.85;5.03]
65 and older		3.26* [2.19;4.85]		4.70* [3.60;6.13]		2.82* [2.09;3.80]		2.83* [1.87;4.29]

**Setting (reference category = Rural)**								
Urban		1.17 [0.83;1.66]		1.04 [0.85;1.27]		1.31* [1.01;1.71]		1.13 [0.81;1.68]

**Marital status (reference category = Married)**								
Widowed		1.13 [0.86;1.49]		1.08 [0.92;1.26]		1.34* [1.09;1.65]		1.51* [1.15;1.97]
Separated/divorced/single		1.12 [0.80;1.61]		0.74 [0.58;1.04]		1.30 [0.93;1.81]		1.42 [0.85;2.37]

**Education (reference category = No formal education)**								
Some formal education		1.32 [0.97;1.78]		1.08 [0.84;1.37]		1.13 [0.80;1.59]		1.48* [1.04;2.09]

**Annual household income (reference category = < PL/2)**								
≥ PL/2-< PL		1.05 [0.85;1.31]		1.19* [1.02;1.39]		1.24* [1.01;1.53]		1.10 [0.84;1.44]
≥ PL-< 2 × PL		1.13 [0.81;1.58]		1.13 [0.93;1.38]		1.21 [0.96;1.52]		1.22 [0.92;1.61]
≥ 2 × PL		1.18 [0.82;1.69]		1.32* [1.04;1.67]		1.25 [0.93;1.68]		1.33 [0.91;1.94]

* = P < 0.05

### Gender and occupation interaction

**Figure 2:**
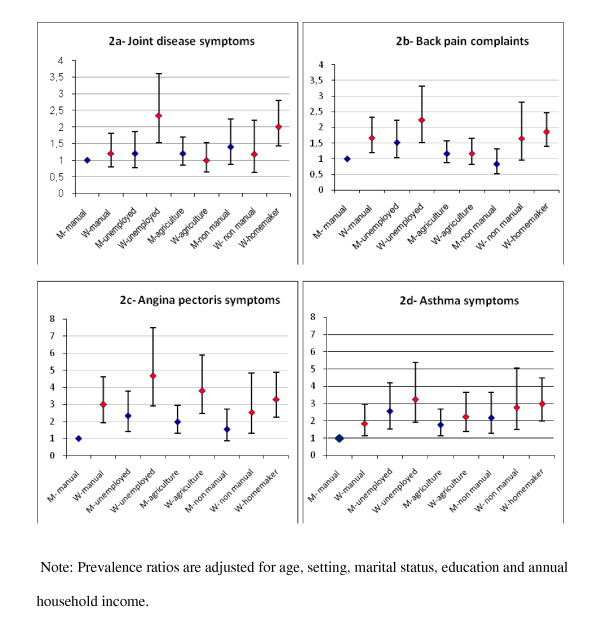
**Prevalence ratios (PR) and confidence intervals (95%) for joint disease symptoms back pain complaints, angina pectoris and asthma symptoms by gender and occupation in the adult population (≥ 18 years old) of Burkina Faso (WHS 2003)**.

Occupation was the only socioeconomic indicator that significantly modified the association between gender and NCD symptoms (Figure 2). Homemakers and unemployed female workers were more apt to report joint disease, back pain, and asthma symptoms than were male manual workers. With the exception of joint disease symptoms in male non-manual and agricultural workers and back pain in male agricultural workers, women had higher prevalence rates of NCDs than did men for all occupation categories, and these differences were greatest among the unemployed.

### Other risk factors

Age was consistently associated with all health conditions as an independent risk factor, whereas urban setting was strongly associated with angina pectoris symptoms (Table 3). Moreover, people who reported having had formal education were more apt to present asthma symptoms than those without any formal education. People living in the second poorest group had significantly higher prevalence of back pain (PR = 1.19; 95% CI = 1.02; 1.39) and angina pectoris (PR = 1.24; 95% CI = 1.01; 1.53) symptoms than did those living in extreme poverty. Back pain symptoms were also significantly more frequent in the wealthiest group (PR = 1.50; 95% CI = 1.06; 2.11) compared to the extremely poor category.

## Discussion

Changing patterns in the causes of mortality and morbidity in low- and middle-income countries constitute one of the most important challenges on the global health agenda. Sub-Saharan Africa is doubly disadvantaged by the persistence of diseases of the pre-transition era--infectious disease conditions still account for two-thirds of deaths in sub-Saharan Africa [21] -- and the concurrent emergence of the heavy burden related to non-communicable diseases. This dual burden of disease [22-24] is a considerable challenge to the countries' health systems. Our analyses show, first, that Burkina Faso, one of the poorest countries on the continent, is already facing the burden of NCDs. The overall prevalences of joint disease and back pain in our study were similar (16.2% [13.5; 19.2] and 24% [21.5; 26.6], respectively) to estimates reported in a comparative study on the prevalence of chronic pain conditions in developing countries [25]. The prevalence of asthma symptoms is also comparable (11.6% [9.5; 14.2]) to overall estimates for Africa [4] and to a previous study in Burkina Faso [5]. Finally, 17.9% [15.8; 20.2] of the adults who participated in the WHS reported angina pectoris-like symptoms; we did not find population estimates in the literature that would help correlate our results with comparable sub-Saharan populations. However, a previous study conducted in an urban setting in Burkina Faso found that the prevalence of hypertension, a known risk factor for angina pectoris, was about 23% among adults [6].

Our observations show a consistent relationship between age and the prevalence of NCD symptoms. However, the significance of this is paralleled by what appears, in terms of health conditions, to be evidence of early aging in the adult population. A markedly high prevalence for all studied conditions was present in young adults, suggesting early onset of NCDs and premature health decline in this population. Joint disease and back pain were unexpectedly high among the youngest, and their frequency increased steeply with age up to a prevalence of over 50% starting in the group aged 55-64 years. These observations converge with those of other studies suggesting that NCDs are both more lethal and developing at earlier ages in sub-Saharan Africa [2,21,26,27]. For example, in a study by Baingana et al., half of cardiovascular disease deaths occurred among people 30 to 69 years of age, which is 10 or more years younger than in more developed regions [27]. Similar trends have been reported in Tanzania [2] and South Asia [28]. In India, 52% of cardiovascular deaths occur before the age of 70 years, whereas the rate in established market economies is only 23% [29]. Rheumatoid arthritis (RA) symptoms seem to occur nearly 10 years earlier in LMICs than in Caucasian populations in the United States and Europe [30,31]. In a South African study, the prevalence of RA among adults 40 years old was significantly higher in native African populations than in people of European origin [31,32]. Asthma in Africa is also more prevalent in adults younger than 40 years of age [4].

Age is the primary predictor of chronic illness survival, and aging explains the growth in prevalence of NCDs globally [7]. However, the aging process does not affect all the world's peoples in the same way [33]. The sources of the differential vulnerability of people living in resource-poor countries and the mechanisms that sustain it are still obscure. The lack of sufficient evidence is exacerbated by the facts that: 1) almost all studies on risk factors for NCDs--particularly cardiovascular diseases--have been carried out in developed countries; 2) it is uncertain to what extent the results of these studies can be directly generalized to the LMIC context [2,24]; and 3) environmental factors could have a significant influence [2,24]. Chronic stress, repeated exposure to infectious diseases and chronic inflammation caused by harsh living conditions might all play a key role in the premature aging of organs and body systems and, ultimately, in the incidence of NCDs [34].

In its recent report on NCDs, WHO [35] reported that poor and disadvantaged populations were more exposed globally to behavioural risks that lead to NCDs. Studies in Asia and Latin America, in particular, have demonstrated the existence of significant associations in LMICs between the prevalence of NCDs and socioeconomic status, especially level of education [35-40]. These associations were not seen in our study in Burkina Faso; education did not appear to have a protective effect on NCDs symptoms, and no pattern emerged from the study of any reported prevalence by income level. In fact, these results are not really surprising in the particular context of Burkina Faso, a sub-Saharan African country with a double burden [2,41,42] of infectious and chronic diseases. In many SSA countries, health inequality remains anchored in social inequality, but is caused also by changes in lifestyles and diet in all social classes [43], particularly among residents of cities and members of advantaged groups. NCDs are thus the product of a complex combination of forces and determinants, and they affect, depending on the circumstances, both disadvantaged and more advantaged classes. This explains the contradictory results reported by studies examining the relationship, in SSA, between socioeconomic status and the presence of NCDs or of their risk factors, such as obesity, hypertension, or metabolic syndrome. Some have reported greater exposure to risk and higher prevalence of NCDs among the poor [35,44,45], while others have reported more pronounced vulnerability among the elite, the middle classes, and city residents [45-49]. WHO and the research community have highlighted the need for more research to better understand the complex interrelationship between NCDs and socioeconomic status [35,43]. There is also evidence that NCDs take a heavier toll on women than on men, and that in this area as in others, women are considerably more exposed to illness and have worse prognoses [28]. It has been suggested that the incidence of chronic diseases in old age is related to life course adversities [50]. Because of their social disadvantage and differential exposure to risk factors [51-53], women are apt to accumulate life course socioeconomic adversities that will be translated into adverse health outcomes. Our observations tend to support the idea that women are subject to greater differential vulnerability than men in terms of NCDs. Gender differences were quite large and significant in our study. Women more often reported symptoms of all four conditions. Differences did not disappear after adjustment for socioeconomic characteristics. When gender and occupation were considered jointly, housewives and unemployed women systematically and significantly remained the group with the highest prevalence rates.

### Limitations

The WHS offer a unique opportunity to obtain population-based estimates of the level and distribution of NCD markers in a resource-poor country such as Burkina Faso. However, because of its cross-sectional design, a cause-effect relationship cannot be inferred. Moreover, measures rely on respondents' answers to a checklist of chronic health conditions [54], and morbidity estimates are ultimately based on self-reported symptoms, an approach whose appropriateness has been extensively debated. A first limitation of this approach resides in the risk of misclassification due to respondents' reporting [55,56]. Memory and social desirability biases can affect peoples' responses [57], and reports might be sensitive to income, literacy or personal expectations [58,59]. The convergence of self-reported data and medical record diagnoses might also vary with the medical conditions investigated [60,61]. A second limitation lies in the common underestimation of the prevalence of medical conditions and risky behaviours [59,62-65]. This underestimation occurs most often in advanced stages of occult disease, or when disease symptomatology is not recognized as representing an unusual threat to a person's health [66]. It most often affects the most socially disadvantaged people, resulting in an underestimation of the social gradient of morbidity [59]. It is also most frequent in the absence of health services utilization [55].

These limitations call for cautious interpretation of surveys based on self-reported measures. However, these limitations should not discourage the use of such surveys. First, despite their limitations, the predictive validity of self-reported symptoms is often acceptable and the observed prevalence estimates are generally reasonable and consistent over time [67], particularly when the survey deals with symptoms that are clear and evocative [60,61]. Others consider WHS self-reported measures to be acceptable for within-country analyses in resource-poor countries [68]. Second, with respect to all surveys, including those of the health examination type, there is still no gold standard approach to estimate the burden of morbidity of the NCDs in this study [56]. Finally, and most importantly, national survey data in both poor [55,56] and industrialized countries [60] provide a unique means of assessing the needs of populations and vulnerable groups, and for planning support services and resource allocation [69]. In fact, health interview surveys have been widely used to measure morbidity in developing countries [55,70] because, in terms of their feasibility, costs and the amount of information they provide, their benefits far outweigh their drawbacks.

## Conclusions

None of the Millennium Development Goals make reference to NCDs. Because of this oversight, governments and the international community have paid little attention to major issues in transforming the post-transition morbidity profiles of African societies. However, these morbidity profiles can be assessed at the population level through the World Health Surveys, from which the amplitude of NCD symptoms can be appreciated. Our work suggests that social inequality extends into the distribution of NCDs among social groups, and supports the thesis of a differential vulnerability in Burkinabè women. It raises the disturbing possibility of an abnormally high rate of premature morbidity that could manifest as a form of premature aging in the adult population. Finally, our work supports the need for sustained commitment to research on population issues related to NCDs in resource-poor countries. The economic and health-related stakes, in terms of both chronic illnesses in general and their premature occurrence in particular, are potentially very high. We believe it is essential, with the help of large population studies, to characterize the distribution of NCDs more precisely across all social and age gradients. Second, more research is necessary to uncover the gender features and understand the sources of women's differential vulnerability to chronic diseases. Third, the question of the alleged premature aging should be at the centre of this research agenda. We need to understand the processes that control the aging of social groups and the underlying causes of the vulnerability of people living in LMICs, and especially the differential vulnerability of specific groups. Finally, health policies should be informed by an estimate of the social burden caused by premature morbidity and its effects on healthcare systems that are struggling to meet their populations' basic needs and are already very fragile.

## Competing interests

The authors declare that they have no competing interests.

## Authors' contributions

MM and SH made substantial contributions to the study's conception, were involved in drafting all sections of the manuscript and gave final approval of the version to be published.

MVZ made substantial contributions to the conception and interpretation of data and was involved in drafting the manuscript.

ÉL was involved in reviewing the literature and helped draft the manuscript.

EEF was involved in data interpretation and in critically reviewing important methodological content of the manuscript.

SK was involved in data interpretation and contextualization and in reviewing the discussion components.

All authors read and approved the final manuscript.

## Pre-publication history

The pre-publication history for this paper can be accessed here:

http://www.biomedcentral.com/1471-2458/12/24/prepub
